# A Synthetic Peptide AWRK6 Alleviates Lipopolysaccharide-Induced Liver Injury

**DOI:** 10.3390/ijms19092661

**Published:** 2018-09-07

**Authors:** Lili Jin, Qiuyu Wang, Hanyu Zhang, Sijia Tai, Hongsheng Liu, Dianbao Zhang

**Affiliations:** 1 School of Life Science, Liaoning University, Shenyang 110036, China; lilijin@lnu.edu.cn (L.J.); qiuyuwang@lnu.edu.cn (Q.W.); zhanghanyu.lnu@gmail.com (H.Z.); taisijia.lnu@gmail.com (S.T.); 2 Research Center for Computer Simulating and Information Processing of Bio-macromolecules of Liaoning Province, Liaoning University, Shenyang 110036, China; liuhongsheng@lnu.edu.cn; 3 Department of Stem Cells and Regenerative Medicine, Key Laboratory of Cell Biology, National Health Commission of China and Key Laboratory of Medical Cell Biology, Ministry of Education of China, China Medical University, Shenyang 110122, China

**Keywords:** AWRK6, synthetic peptide, lipopolysaccharides (LPS), liver injury, apoptosis

## Abstract

During lipopolysaccharide (LPS)-induced sepsis, the liver plays central roles in toxins phagocytosis and clearance to protect the whole body. The liver cells were constantly irritated by LPS which leads to liver injury. While most anti-LPS agents showed little clinical activity against LPS-induced liver injury. Here, the protective effects of the synthetic peptide AWRK6 against LPS-induced liver injury have been investigated in vivo and in vitro. In mice liver homogenate, LPS administration elevated ALT (alanine aminotransferase), iNOS (inducible nitric oxide synthase) and repressed SOD (superoxide dismutase) activities and these changes were remarkably reversed by AWRK6. Histologically, AWRK6 effectively alleviated the histological changes and repressed LPS-induced neutrophils infiltration. By TUNEL assay on liver sections, AWRK6 was proven to inhibit apoptosis induced by LPS in mice livers, which was also verified by the protein levels of cleaved-caspase 9, Bax and Bcl-2. In addition, by in vitro study using HepG2 cells, AWRK6 was found to recover the LPS-reduced cell viability and reduce LPS-induced apoptosis. For mechanisms, AWRK6 was demonstrated to alleviate the LPS-induced phosphorylation of ERK, JNK and p38 MAPK, indicating the involvement of MAPKs in the protection of AWRK6 against liver injury. In summary, we have found the synthetic peptide AWRK6 as a promising novel agent for LPS-induced liver injury, by inhibiting cell apoptosis through MAPK signaling pathways, which might bring new strategies for the treatment of acute and chronic liver injuries.

## 1. Introduction

Lipopolysaccharides (LPS), a major component of gram-negative bacteria outer membrane, can induce sepsis and the mortality exceeds 20% [1]. During sepsis, the liver plays central roles in toxins phagocytosis and clearance to protect the whole body and the liver itself [2]. The liver cells were constantly irritated by LPS, leading to liver injury [3,4]. Most anti-LPS agents, such as antibiotics and antibodies, showed little clinical activity against liver injury, such as the polymyxin antibiotics colistin and polymyxin B (PMB), which became clinically available for LPS neutralization in the 1950s before being shortly restricted due to its toxicity [5,6]. Nowadays, the drug-resistant bacteria and LPS release caused by the use of large amounts of antibiotics make the problems more serious [7]. Therefore, it is urgent to develop novel agents and strategies against LPS, especially for liver injury.

Antimicrobial peptides are important group of innate immunity effectors with broad-spectrum antibiosis, found among almost all classes of animals [8]. Recent studies revealed that antimicrobial peptides LL-37 and β-defensin could protect septic mice from death by neutralizing LPS [9,10]. In our previous studies, a novel antimicrobial peptide dybowskin-2CDYa (SAVGRHSRRFGLRKHRKH, GenBank: ACF08009.1) had been discovered and characterized [11]. The peptide was further modified by replacing Arg with Lys to improve its antimicrobial activity and stability, into the sequence of SWVGKHGKKFGLKKHKKH, which was named AWRK6 [11,12]. The 18-amino acids cationic polypeptide AWRK6 showed effective antimicrobial activity and in the very recent study, we found AWRK6 as a novel potential agent against LPS-induced inflammatory responses [13]. In mice with LPS-induced endotoxemia, AWRK6 prevented LPS-induced lethality by blocking LPS binding to LBP and attenuating the release of IL-1β, IL-6 and TNF-α. It presented promotion effect on the healing of liver injury in histopathological analysis [13], while the detailed functions and mechanisms of AWRK6 against LPS-induced liver injury were still unknown.

In this study, we have investigated the protective effects of AWRK6 against LPS-induced liver injury in mice and HepG2 liver cells. Liver cell apoptosis and MAPK pathways activation were examined to access the fundamental mechanisms. AWRK6 was found as a novel agent for LPS-induced liver injuries, though inhibiting liver cell apoptosis.

## 2. Results

### 2.1. AWRK6 Relieved LPS-Induced Liver Injury in Mice

**Figure 1:**
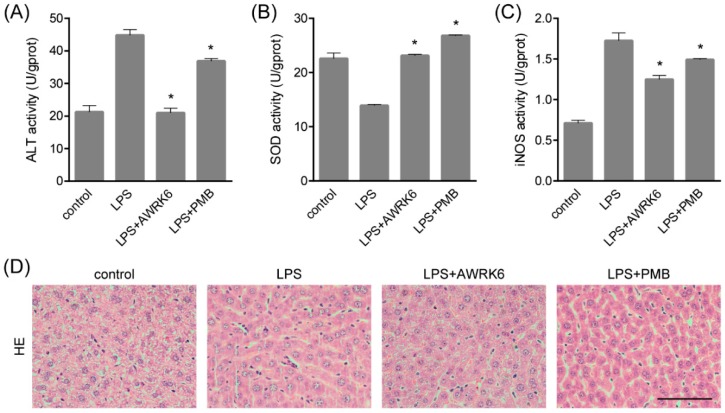

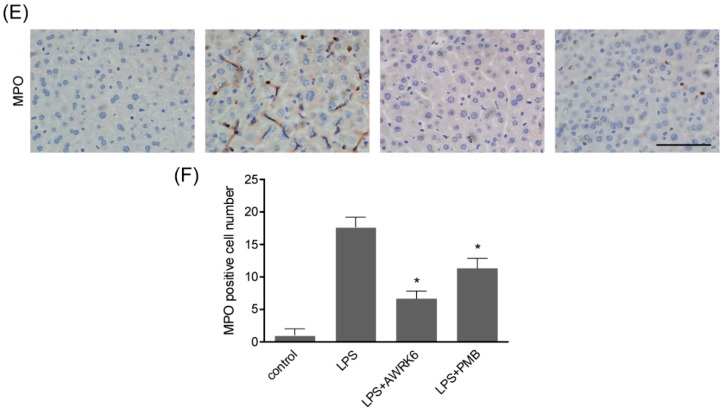
AWRK6 relieved lipopolysaccharide (LPS)-induced liver injury in mice. The mice were administrated with LPS (50 mg/kg) by intraperitoneal injection. AWRK6 (10 mg/kg) or PMB (10 mg/kg) was administered intraperitoneally 1 h after LPS and an equal volume of sterile saline was used as a negative control. (**A**) The protective effect of AWRK6 on enhanced ALT activity in LPS-treated mice liver, assayed by Alanine aminotransferase Assay Kit. (**B**) The effect of AWRK6 on SOD activity in LPS-treated mice liver assayed by Superoxide Dismutase Assay Kit. (**C**) The effect of AWRK6 on iNOS activity in LPS-treated mice liver, assayed by Nitric Oxide Synthase Assay Kit. (**D**) Histologic changes of liver tissues upon LPS and AWRK6 treatment, stained with HE. (**E**) Micrographs of liver sections stained with MPO antibody. (**F**) The analysis of liver sections stained with MPO was carried out using ImageJ. * *p* < 0.05 compared with the LPS groups. Scale bar indicates 100 μm.

In order to investigate the protective effects of AWRK6 against LPS-induced liver injury, mice model was constructed by LPS (50 mg/kg) administration through intraperitoneal injection. AWRK6 (10 mg/kg) or PMB (10 mg/kg) was administered intraperitoneally 1 h after LPS, an equal volume of sterile saline was used as a negative control. To evaluate the liver injury, several hepatic enzymes in liver homogenate were examined. As presented in Figure 1A–C, LPS administration significantly elevated ALT (alanine aminotransferase), iNOS (inducible nitric oxide synthase) and repressed SOD (superoxide dismutase) activities and these changes were remarkably reversed by AWRK6 treatment for 24 h, indicating the protective effects of AWRK6 against LPS-induced liver damage. Besides, AWRK6 showed better effect than the positive control PMB, which could neutralize LPS efficiently [5]. Histologically, LPS treatment decreased cell density and enlarged cell gap, compared with the blank control. AWRK6 effectively alleviated the histological changes and repressed LPS-induced neutrophils infiltration, which was MPO (myeloperoxidase) positive (Figure 1D–F). These results suggested that AWRK6 could promote the functional recovery of liver injury induced by LPS, which was comparable to PMB.

### 2.2. AWRK6 Inhibited LPS-Induced Liver Cell Apoptosis in Mice

**Figure 2:**
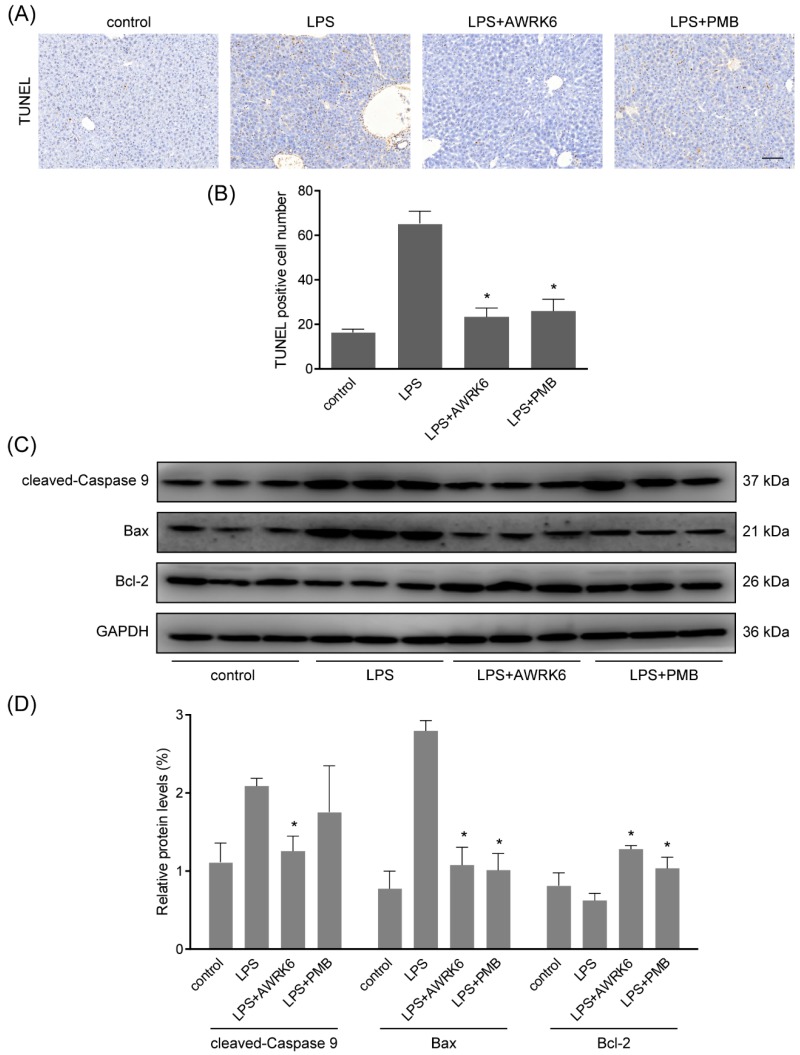
AWRK6 inhibited LPS-induced apoptosis in mice liver. (**A**) AWRK6 (10 mg/kg) treatment for 24 h reduced DNA fragmentation induced by LPS (50 mg/kg), assayed by TUNEL assay. (**B**) The results of TUNEL assay were analyzed by ImageJ. (**C**) The protein levels of cleaved-caspase 9, BAX and Bcl-2 were analyzed by western blotting. (**D**) The quantification of western blotting results was carried out using ImageJ. * *p* < 0.05 compared with the LPS groups. Scale bar indicates 100 μm.

By TUNEL assay (terminal deoxynucleotidyl transferase-mediated dUTP-biotin nick end labeling), fragmented DNA generated during apoptosis was stained with Biotin-dUTP and Streptavidin-HRP. The liver sections showed enhanced apoptotic cells in LPS-treated group and AWRK6 treatment significantly inhibited liver cell apoptosis in mice liver, which was more effective than PMB (Figure 2A,B). Further, the key regulators of apoptosis including cleaved-caspase 9, Bax and Bcl-2 were detected using western blotting. As shown in Figure 2C,D, cleaved-caspase 9 and Bax were enhanced and Bcl-2 was reduced upon LPS treatment. AWRK6 treated group showed similar levels of cleaved-caspase 9, Bax as the blank control and enhanced Bcl-2. These results demonstrated that AWRK6 administration could inhibit LPS-induced liver cell apoptosis to protect liver injury in mice model.

### 2.3. AWRK6 Inhibited LPS-Induced Liver Cell Apoptosis in HepG2 Cells

**Figure 3:**
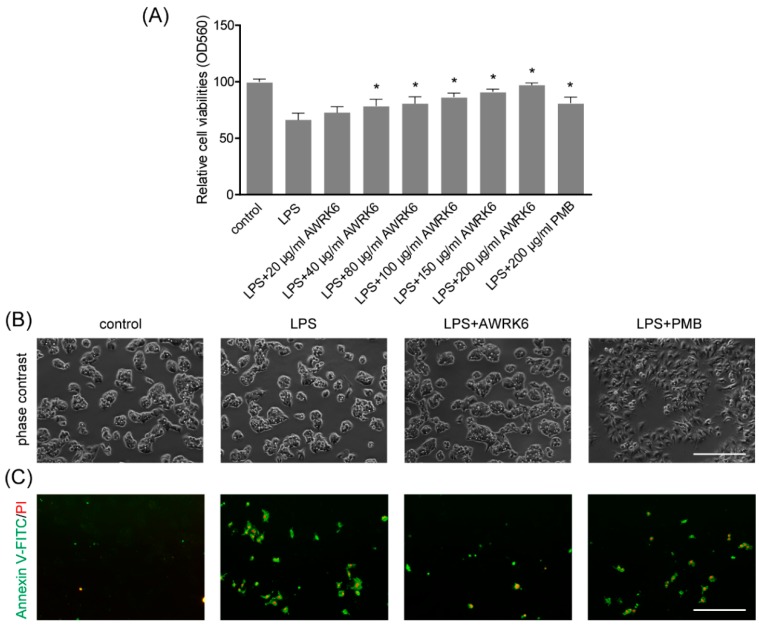

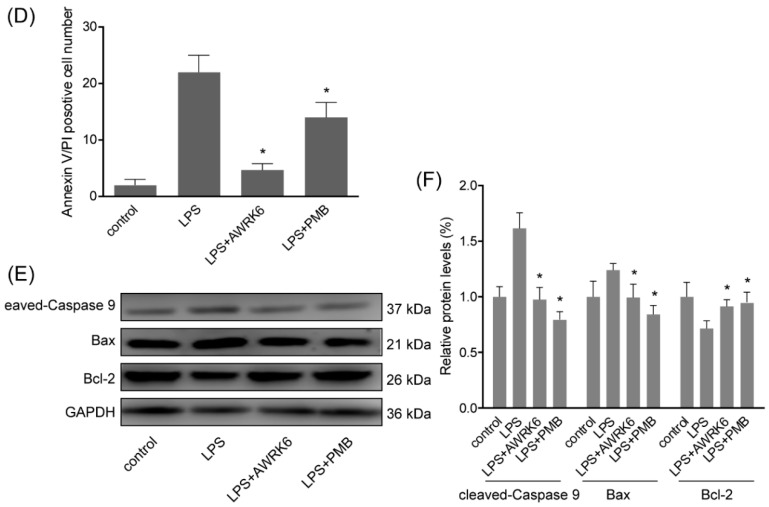
AWRK6 inhibited LPS-induced liver cell apoptosis in HepG2 cells. (**A**) The viabilities of HepG2 liver cells treated with LPS (40 μg/mL) with/without AWRK6 for 24 h, examined by MTT assay. (**B**) The cells treated with LPS and AWRK6 (200 μg/mL) were observed under phase contrast microscope. (**C**) The cell apoptosis was detected by Annexin V-FITC/PI staining followed by fluorescence microscopy. (**D**) The apoptotic cell number in the results of Annexin V-FITC/PI staining was analyzed by ImageJ. (**E**) The protein levels of cleaved-caspase 9, BAX and Bcl-2 were analyzed by western blotting. (**F**) The results of western blotting were quantified using ImageJ. Bar indicates 100 μm. * *p* < 0.05 compared with the LPS groups.

To gain more insight into the consequences of AWRK6 treatment on liver cell, in vitro experiments were carried out in HepG2 liver cell. HepG2 cells were treated with 40 μg/mL LPS with/without AWRK6 at different concentrations. PMB at 200 μg/mL was used as a positive control. The cell viabilities were determined using MTT assay. As shown in Figure 3A, LPS (40 μg/mL for 24 h) stimulation significantly reduce the dehydrogenase activity, which is directly proportional to the number of living cells. And when the LPS-treated cells were incubated with AWRK6 (20, 40, 80, 100, 150 and 200 μg/mL), the cell viability was recovered in a concentration dependent manner, compared with the control group. Under phase contrast microscope, the cell morphology showed no significant change upon the treatment with LPS and AWRK6 (200 μg/mL), while in PMB (200 μg/mL) treated group, the cells were more spread, indicating the potential toxicity of PMB (Figure 3B). By Annexin V-FITC/PI Staining, the early (Annexin V+/PI−) and late (Annexin V+/PI+) apoptotic cells were observed under fluorescence microscopy. In the results shown in Figure 3C,D, the LPS-induced apoptotic cell number was reduced after AWRK6 treatment for 24 h, which was close to the control. While PMB presented weaker effect. Also, the protein levels of cleaved-caspase 9, Bax and Bcl-2 were analyzed by western blotting. The elevated cleaved-caspase 9, Bax and repressed Bcl-2 could be reversed by AWRK6 treatment, which was consistent with the in vivo results (Figure 3E,F). These results demonstrated that AWRK6 could relieve apoptosis induced by LPS in liver cells, providing a potential apoptosis inhibitor for LPS-induced liver injury.

### 2.4. MAPKs Were Involved in the Protection of AWRK6 against Liver Injury

**Figure 4:**
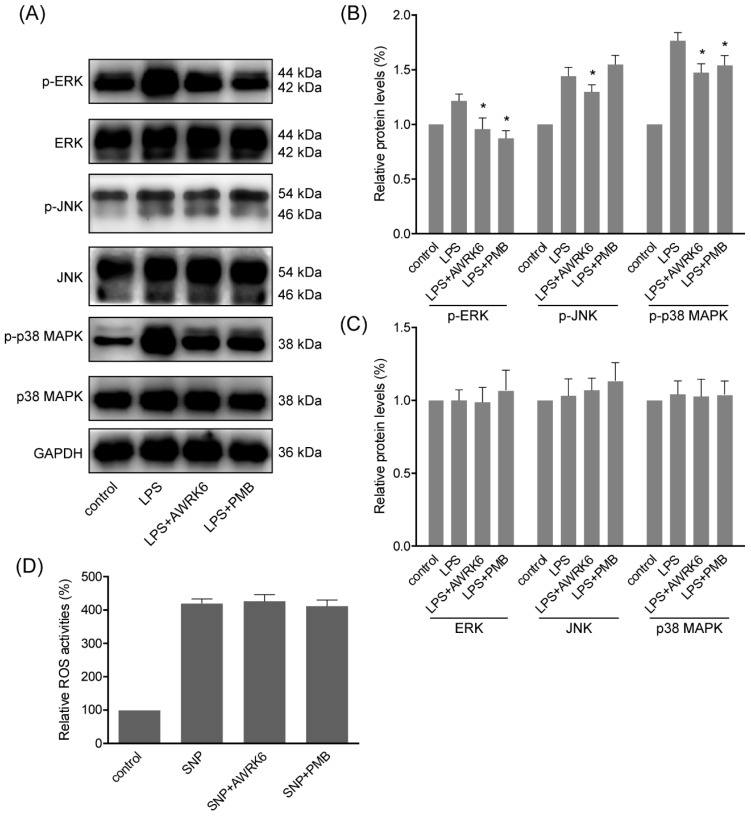
MAPKs were involved in the protection of AWRK6 against liver injury. (**A**) The phosphorylation and total proteins of MAPKs including ERK, JNK and p38 MAPK in HepG2 cells were analyzed by western blotting, following the treatment with LPS (40 μg/mL) and AWRK6 (200 μg/mL) for 2 h. (**B**) The phosphorylation of MAPKs was analyzed by ImageJ. (**C**) The total proteins of MAPKs were analyzed by ImageJ. (**D**) The ROS level in HepG2 cells treated by 400 μM sodium nitroprusside (SNP) with/without AWRK6 at 200 μg/mL for 6 h, assayed by Reactive Oxygen Species Assay Kit. The results were presented as fluorescence intensity, percent of control. * *p* < 0.05 compared with the LPS groups.

During LPS-induced inflammatory response and cell apoptosis, MAPK (mitogen-activated protein kinases) pathways are generally activated to induce pro-apoptotic factors and active NFκB pathway, which is in direct relation to iNOS expression and cytokines secretion [14]. To elucidate MAPKs activation during the protection of AWRK6 against LPS-induced apoptosis, we assessed the phosphorylation of ERK (extracellular signal-regulated kinase), JNK (c-Jun N-terminal kinase) and p38 MAPK following treatment with LPS (40 μg/mL) and AWRK6 (200 μg/mL) for 2 h. As shown in Figure 4A–C, the phosphorylation of ERK, JNK and p38 MAPK were markedly elevated by the stimulation with LPS. And upon the treatment with AWRK6, the phosphorylation of ERK, JNK and p38 MAPK were significantly inhibited. Meanwhile, the total protein of ERK was inhibited by LPS and relived by AWRK6, while JNK and p38 MAPK showed no significantly changes upon AWRK6 treatments, relative to GAPDH. These data proved that the LPS-induced activation of MAPK pathways including ERK, JNK and p38 MAPK could be inhibited by AWRK6 in LPS-challenged HepG2 liver cells, indicating the involvement of MAPK pathways in the protection of AWRK6 against liver injury.

Considering that LPS is a complicate molecule and it could interact with LBP, while it is not clear whether AWRK6 interact with membrane receptors or has any tentative intracellular target. The effects of AWRK6 against sodium nitroprusside induced ROS (Reactive Oxygen Species) in HepG2 cells were analyzed using DCFH-DA. The HepG2 cells were treated by 400 μM sodium nitroprusside with/without AWRK6 at 200 μg/mL for 6 h. The intracellular ROS levels were analyzed by Reactive Oxygen Species Assay Kit. In Figure 4D, the results were presented as fluorescence intensity, percent of control. The ROS level was significantly elevated by sodium nitroprusside and AWRK6 showed no significant effect on the ROS level. Based on the previous results, the protective effects of AWRK6 against LPS in HepG2 cells might be mainly due to the extracellular neutralization of LPS.

## 3. Discussion

LPS is a major structural and functional component of the gram-negative bacterial outer membranes, it could be released during cell death, cell division and the treatment with antibiotics [15]. LPS in blood leads to endotoxemia with liver injury which affects liver function [16]. LPS directly induces liver injury through activating inflammatory cells such as neutrophils and inducing chemical mediators including superoxide and nitric oxide [17,18]. The stimulation with LPS on immune cells activation, inflammation and apoptosis plays important roles in acute and chronic liver diseases [19]. Until now, there is no safe and effective anti-LPS drug. The discovery of LPS neutralization properties of antimicrobial peptides brought us a novel way. The peptide S3 which was derived from Sushi3 domain of Factor C was found to bind with LPS directly [20]. Magainin 2 binding with LPS by its α-helical structure caused the leakage of liposomes [21]. Melittin presented interaction with lipopolysaccharide to inhibit lipopolysaccharide-induced pro-inflammatory response in macrophage cells [14]. However, antimicrobial peptides are still rare in daily clinical practice due to their efficiency and side-effects. rBPI21, a fragment of neutrophils BPI protein with a strong affinity for LPS, is beneficial in decreasing complications of meningococcal disease, while it presented no significant differences in mortalities of placebo and treated groups in clinical trial [22]. A phase II/III study of the safety and efficacy of talactoferrin in patients with severe sepsis found a higher 28-day mortality rate in the talactoferrin group, the reason remains unclear [23]. Thus, newly discovered and developed antimicrobial peptides with better efficacy and decreased toxicity are essential to improve therapy against LPS. Recently, we have found the inhibitory properties of AWRK6 against LPS-induced inflammatory responses in mice [13]. Here, we extended it to the liver protective roles of AWRK6 in vivo and in vitro. In this study, the liver tissue of mice challenged with LPS showed remarkably morphology changes and neutrophils infiltration. The liver injury marker ALT and the immune response indicator iNOS were enhanced by LPS administration. We found AWRK6 as a liver protective agent which could reverse these changes during liver injury induced by LPS, consistent with the in vitro results that AWRK6 incubation alleviated LPS-induced viability reduction of liver cells. These data indicated AWRK6 was a potential agent against LPS-induced liver injury for functional and structural recovery.

Apoptosis is a programmed cell death process involved in liver injury induced by LPS, it leads to characteristic changes including chromosomal DNA fragmentation, phosphatidylserine on the cell surface and caspase activation [24,25,26,27]. Here, the DNA fragmentation in mice liver tissue was detected via TUNEL assay, AWRK6 treatment showed remarkably reduction in apoptotic liver cell number. The protective effects of AWRK6 against liver cell apoptosis were also verified in vitro by phosphatidylserine detection using Annexin V-FITC/PI staining. During cell apoptosis, Bcl-2 and Bax are key regulators [28,29]. Bcl-2 on the outer membrane of mitochondria plays key roles in promoting cell survival and inhibiting pro-apoptotic proteins, while Bax could form a heterodimer with Bcl-2 to induce apoptotic process [30]. Bax contributed to the damage of the mitochondrial membrane, promoting the permeabilization and release of cytochrome C, which could activate caspase 9 and act as an important signal in apoptosis [31]. In LPS-administrated mice liver, cleaved-caspase 9 and Bax were enhanced and Bcl-2 was reduced, while AWRK6 could significantly reverse these apoptotic changes, as further proved in vitro by western blotting. These experiments provided evidences revealing the anti-apoptosis effects of AWRK6 in LPS-induced liver injury in vivo and in vitro.

LPS could interact with LPS-binding protein (LBP) and initiate intracellular signaling through Toll-like receptor 4 (TLR4), which could active different signals, such as MAPKs and NFκB, leading to cell apoptosis and the secretion of pro-inflammatory cytokines such as IL-1β, IL-6 and TNF-α [13,14,32,33]. The MAPKs are a group of serine-threonine protein kinases playing important roles in extracellular signaling transduction to regulate cell proliferation, differentiation and apoptosis [34]. The regulation of MAPKs in mice liver was involved in liver injury [2,35,36]. The MAPK family members including ERK, JNK and p38 MAPK. ERK is often activated by cytokines and cell stress involved in cell division and apoptosis; JNK is a key regulator in response to heat shock, oxidative stress and DNA damage; and P38 MAPK was activated by inflammatory cytokines and environmental stresses [37]. LL-37 could suppress LPS-induced keratitis in mice by attenuating phosphorylation of MAPKs [38]. The inhibition of MAPK pathways by melittin was involved in LPS-induced inflammatory reaction [39]. In our previous study, AWRK6 was found to attenuate the serum levels of LPS, IL-1β, IL-6 and TNF-α in endotoxemic mice by inhibiting NFκB activation [13]. Here, the phosphorylation of ERK, JNK and p38 MAPK were analyzed. The LPS-induced phosphorylation of ERK, JNK and p38 MAPK were repressed by AWRK6 treatment in liver cells, indicating the involvement of MAPKs in the protection of AWRK6 against LPS-induced liver cell apoptosis.

In the present study, we have found that the synthetic peptide AWRK6 was a promising novel agent for LPS-induced liver injury, by inhibiting the cell apoptosis through MAPK signaling pathways. These findings might bring new strategies for the treatment of acute and chronic liver injuries.

## 4. Materials and Methods

### 4.1. Mice Model

Female Kunming mice (18–22 g) were obtained from Experimental Animal Research Center of Shenyang Medical College (Shenyang, China) and housed in individual cages with free access to food and water. LPS (50 mg/kg, L2880, Sigma, Shanghai, China) were administrated by intraperitoneal injection. AWRK6 (10 mg/kg, synthesized by GL Biochem, Shanghai, China) or PMB (10 mg/kg, YZ-130313, Solarbio, Beijing, China) was administered 1 h after LPS. An equal volume of sterile saline was used as a negative control. The procedures were approved by the Ethics Committee of Liaoning University (20150011, 23 February 2015) and conducted according to the Care and Use of Laboratory Animals.

### 4.2. ALT, SOD and iNOS Assay

The liver tissues were grinded into tissue homogenates on the ice. ALT was detected using the Alanine aminotransferase Assay Kit (C009-2, Nanjing Jiancheng Bioengineering Institute, Nanjing, China). SOD was detected by Superoxide Dismutase Assay Kit (A001-3, Nanjing Jiancheng Bioengineering Institute). iNOS was detected using Nitric Oxide Synthase Assay Kit (A014-1-1, Nanjing Jiancheng Bioengineering Institute). The absorbance was determined using an iMARK microplate reader (Bio-Rad, Hercules, CA, USA).

### 4.3. Histopathological Examination

The liver tissues were fixed in 10% formalin and embedded in paraffin. The tissue sections (5 μm) were made and stained by hematoxylin and eosin (G1120, Solarbio) or MPO antibody (RAB-0379, Maxim, Fuzhou, China) with UltraSensitive SP (Mouse/Rabbit) IHC Kit (KIT-9710, Maxim) according to the manufacturer’s instructions. Histopathology images were obtained under a light microscopy (CK31, Olympus, Tokyo, Japan).

### 4.4. TUNEL Assay

Cell apoptosis in liver tissues was detected using TUNEL assay (C1091, Beyotime, Shanghai, China), according to the manufacturer’s instructions. The liver tissue sections were dewaxed and treated with proteinase k, followed by incubation with TUNEL detection buffer in the dark for 1 h at 37 °C. The sections were observed and photographed using an Olympus CK31 microscope.

### 4.5. Western Blotting

The total protein was prepared by RIPA lysis buffer (R0010, Solarbio) and quantified by BCA assay (MA0082, Meilunbio, Dalian, China). Equal amounts of protein were separated on 10% SDS-PAGE and transferred to PVDF membranes. The membranes were incubated overnight at 4 °C with antibodies against cleaved-caspase 9 (1:2000, 9509s, CST, Shanghai, China), Bax (1:2000, TA810334, OriGene, Beijing, China), Bcl-2 (1:2000, UM870117, OriGene), GAPDH (1:8000, TA-08, ZSGB Bio, Beijing, China), phospho-ERK (1:1500, 4370T, CST), ERK (1:1500, 4695T, CST), phosphor-JNK (1:1500, 4668T, CST), JNK (1:1500, 9252T, CST), phosphor-p38 MAPK (1:1500, 4511T, CST) and p38 MAPK (1:1500, 8690T, CST), followed by incubation with HRP-conjugated secondary antibodies (1:8000, ZDR-5306 and ZDR-5307, ZSGB Bio). Protein bands were visualized by Meilunbio^®^ fg super sensitive ECL luminescence reagent (MA0186, Meilunbio) and imaged on a DNR MicroChemi chemiluminescence detection system (DNR, Israel). The protein bands were analyzed using ImageJ software.

### 4.6. Cell Culture

The human liver HepG2 cells were obtained from Procell (CL-0103, Wuhan, China). The cells were cultured in DMEM with 10% fetal bovine serum (SA212.02, CellMax, Beijing, China) and 1% Penicillin streptomycin (P1400, Solarbio) at 37 °C in an incubator. The cells were treated with LPS, AWRK6, PMB and Sodium Nitroprusside (S0015, Beyotime) at the indicated concentrations and subsequent experiments were carried out.

### 4.7. Cell Viability Assay

The cell viability was assayed using MTT (MB4698, Meilunbio). The cells were seeded into 96-well plates at 4000 cells per well and incubated overnight. After indicated treatments, 10 μL MTT reagent was added to each well and incubated for 4 h. The absorbance at 560 nm was detected by iMARK microplate reader.

### 4.8. Annexin V-FITC/PI Staining

The cell apoptosis was detected using FITC Annexin V Apoptosis Detection Kit I (556547, BD Biosciences). The cells were cultured and treated in 6-well plates. Annexin V-FITC and PI staining were carried out according to the manufacturer’s instruction. The cells were analyzed by fluorescence microscopy Observer A1 (Zeiss, Oberkochen, Germany).

### 4.9. Reactive Oxygen Species Assay

The HepG2 cells were seeded in 96-well black plates for 12 h and treated by 400 μM sodium nitroprusside (S0015, Beyotime) with/without AWRK6 at 200 μg/mL for 6 h. The intracellular ROS levels were analyzed with a Reactive Oxygen Species Assay Kit (S0033, Beyotime) using a Tecan Infinite M200 microplate reader (Tecan, Männedorf, Switzerland) at excitation and emission wavelengths of 488 and 525 nm respectively.

### 4.10. Statistical Analysis

The data were presented as mean ± SD and analyzed by *t* test and one-way ANOVA, followed by multiple comparisons, using GraphPad Prism 6 software (GraphPad Software, Inc., La Jolla, CA, USA). *p* < 0.05 Was considered statistically significant.
